# Predictive features of chronic kidney disease in atypical haemolytic uremic syndrome

**DOI:** 10.1371/journal.pone.0177894

**Published:** 2017-05-18

**Authors:** Matthieu Jamme, Quentin Raimbourg, Dominique Chauveau, Amélie Seguin, Claire Presne, Pierre Perez, Pierre Gobert, Alain Wynckel, François Provôt, Yahsou Delmas, Christiane Mousson, Aude Servais, Laurence Vrigneaud, Agnès Veyradier, Eric Rondeau, Paul Coppo, French Thrombotic Microangiopathies Reference Centre

**Affiliations:** 1 Centre de Reference des Microangiopathies Thrombotiques, Hôpital Saint Antoine, AP-HP, Paris, France; 2 Urgences Néphrologiques et Transplantation Rénale, Hôpital Tenon, AP-HP, Paris, France; 3 Service de Néphrologie, Hôpital Bichat, AP-HP, Paris, France; 4 Service de Néphrologie-immunologie clinique, Hôpital Rangueil, Toulouse, France; 5 Service de Réanimation Médicale, Centre Hospitalier Universitaire de Caen, Normandy, France; 6 Service de Néphrologie - Médecine Interne, Hôpital Sud, Amiens, France; 7 Service de Néphrologie-Immunologie, Service de Réanimation, CHU Brabois, Nancy, France; 8 Service de Médecine Interne et Néphrologie, Hôpital Général Henri Duffaut, Avignon, France; 9 Service de Néphrologie, Hôpital Maison Blanche, Reims, France; 10 Service de Néphrologie, Hôpital Albert Calmette, Lille, France; 11 Service de Néphrologie-Transplantation-Dialyse, CHU de Bordeaux, Bordeaux, France; 12 Service de Néphrologie, Dijon, France; 13 Service de Néphrologie, Hôpital Necker-Enfants Malades, AP-HP, Paris, France; 14 Service de Médecine interne, Néphrologie et Médecine vasculaire, Centre hospitalier de Valenciennes, Valenciennes, France; 15 Service d'Hématologie Biologique, Hôpital Lariboisière, AP-HP, Paris, France; 16 Service d’Hématologie, Hôpital Saint Antoine, AP-HP, Paris, France; Istituto Di Ricerche Farmacologiche Mario Negri, ITALY

## Abstract

Chronic kidney disease (CKD) is a frequent and serious complication of atypical haemolytic uremic syndrome (aHUS). We aimed to develop a simple accurate model to predict the risk of renal dysfunction in aHUS based on clinical and biological features available at hospital admission. Renal function at 1-year follow-up, based on an estimated glomerular filtration rate < 60mL/min/1.73m^2^ as assessed by the Modification of Diet in Renal Disease equation, was used as an indicator of significant CKD. Prospectively collected data from a cohort of 156 aHUS patients who did not receive eculizumab were used to identify predictors of CKD. Covariates associated with renal impairment were identified by multivariate analysis. The model performance was assessed and a scoring system for clinical practice was constructed from the regression coefficient. Multivariate analyses identified three predictors of CKD: a high serum creatinine level, a high mean arterial pressure and a mildly decreased platelet count. The prognostic model had a good discriminative ability (area under the curve = .84). The scoring system ranged from 0 to 5, with corresponding risks of CKD ranging from 18% to 100%. This model accurately predicts development of 1-year CKD in patients with aHUS using clinical and biological features available on admission. After further validation, this model may assist in clinical decision making.

## Introduction

Haemolytic uremic syndrome (HUS) is a thrombotic microangiopathy (TMA) characterized by a prominent renal impairment resulting from fibrin and/or platelet thrombi in kidney microvasculature and a detectable von Willebrand factor-cleaving metalloproteinase ADAMTS13 activity (typically > 20%) [1]. The most common form of HUS is caused by a prodromal bacterial infection (a shigatoxin-producing *Escherichia coli*–STEC) [2]. In the absence of STEC, HUS is termed atypical. Atypical HUS (aHUS) results directly from damage caused by the uncontrolled activation of the alternative complement pathway leading to excessive complement activation on cell membranes [3]. Mutations in the complement system genes have been reported in 50% to 60% of aHUS. The mutations identified impair regulation in the alternative pathway at the level of the C3 convertase. These mutations involve genes encoding for complement factor H (CFH), membrane cofactor protein (MCP/CD46), complement factor I (CFI), complement factor B (CFB) and C3. Anti-CFH antibodies have also been reported [4,5]. Mutations in patients with aHUS have also been identified in the coagulation pathway (thrombomodulin and plasminogen), linking these two pathways to the development of aHUS. Recently, mutations in the diacylglycerol kinase ε gene have been found to cause HUS-like disease in paediatric patients < 1-yo [6].

Prognosis of aHUS is poor with a global mortality rate of 25% and a frequent progression to chronic kidney disease (CKD). Over half of patients develop end-stage renal disease (ESRD) after the first aHUS presentation and quality of life is consequently significantly impacted [4]. However, the disease is heterogeneous and unpredictable and the clinical characteristics of patients with aHUS and the risk of TMA after renal transplant seems to vary based on specific complement mutations and environmental factors. Patients with mutations of CFH have the poorest prognosis with an up to 79% mortality or ESRD rate at 3 years and extra-renal lesions in 20% of cases, whereas in patients with MCP mutations mortality/ESRD rate is 20% [4]. However, the assessment of aHUS prognosis from complement mutational analysis has limitations. First, more than half of patients with poor renal outcome have no identifiable complement abnormalities [4]. Moreover, genetic analysis in aHUS still requires skill and time. Therefore, this exploration usually falls within some highly expert international laboratories and the results are not rapidly available for clinical practice.

For years, treatment of aHUS mainly consisted of plasma exchange (PE) and dialysis with poor efficacy. Recently, the complement system blocker eculizumab (Soliris^®^, Alexion), a humanized monoclonal immunoglobulin G antibody that targets the C5 fraction of complement [7], remarkably improved response rates with a complete reversal of TMA features in more than 70% of PE-dependent or -refractory aHUS, and a significant and continued improvement in renal function with dialysis discontinuation in 80% of cases [8]. Of note, eculizumab also provided improvement in patients with no evidence of complement abnormalities [8,9]. Moreover, studies demonstrated that earlier initiation of eculizumab therapy was associated with a greater improvement in kidney outcome [8–10]. Therefore, eculizumab is becoming the standard of care in aHUS [8,9,11]. However, treatment with eculizumab has limitations. First, it exposes patients to a significant risk of invasive meningococcal infection despite vaccination and antibioprophylaxis. Second, current recommendations state that eculizumab should be pursued for an undetermined period, which raises cost concerns. Therefore, it is crucial to determine rapidly patients who should benefit the most from complement blockers, and it remains still unclear if all patients with features consistent with aHUS, i.e., a microangiopathic haemolytic anaemia with a detectable ADAMTS13 activity, should be systematically treated with complement blockers for a long time [11].

Here, we searched to determine risk factors on admission associated with adverse renal outcome at 1-year in a cohort of adults with a diagnosis of aHUS and treated with only PE. The early identification of patients prone to develop CKD could indeed help selecting those who may benefit from complement blockers frontline instead of PE.

## Patients and methods

### Study design

Adult (>18-yo) patients of our National Registry recruited mostly from nephrology and intensive care units from 17 leading French centres were studied from October, 2000 to June, 2014 [12]. aHUS was diagnosed in patients with thrombocytopenia associated with microangiopathic haemolytic anaemia, acute renal failure and ADAMTS13 activity ≥20%. Patients with underlying diseases impacting possibly aHUS prognosis were excluded: HIV infection, cancer and/or chemotherapy, transplantation, connective tissue diseases and malignant hypertension. We also excluded all patients with a STEC infection, and patients with an infection attributed to *Escherichia coli* or an asymptomatic colonization, even though no shigatoxin was detected. For all patients, the final diagnosis of aHUS was confirmed by the principal investigator (MJ) and at least one expert investigator (PC and/or ER).

We systematically captured clinical features on admission, at discharge and when available at three, six and twelve months. Renal function was assessed by estimating the glomerular filtration rate (eGFR) with the Modification of diet in renal disease equation (MDRD). ADAMTS13 activity and complement analysis on plasma were performed on admission as previously reported [5,13]. Genetic analysis of aHUS was mainly performed in patients with no evidence of infectious triggering factor [5].

Treatment of aHUS was derived from the recommendations used for thrombotic thrombocytopenic purpura (TTP) [12]. Briefly, PE with plasma was started daily immediately after HUS diagnosis. The volume exchanged was 1.5 X the predicted plasma volume until remission. Complete response was defined as full resolution of renal failure or stabilization of renal function in patients considered with CKD and recovery of normal platelet count (≥ 150 X 10^3^/μL). Relapse was defined as the reappearance/worsening of renal failure and/or thrombocytopenia after remission [11,12].

Informed consent was obtained from all patients. Because our study was retrospective and based on data involving standard care assessment, a verbal consent was obtained and reported in the patient’s record, in accordance with the local ethical committee. All analysed data were anonymised. This study was approved by our institutional review board in accordance with the Declaration of Helsinki (CPP Ile-de-France V, Hôpital Saint Antoine, Paris), and the French Data Protection Authority (“Commission Nationale Informatique et Libertés”, CNIL, authorization n°911539, and “Comité consultatif sur le traitement de l’information en matière de recherche dans le domaine de la santé”, CCTIRS, authorization n°11.537, Paris, France).

### Statistical analysis

CKD was defined by an eGFR below 60 mL/min/1.73m^2^ (stage 3a to 5 of CKD) (S1 Table) [14]. Univariate screening for predictors of CKD at one year used the Fisher’s test or Student’s t-test. Variables significantly associated with outcome in the univariate analysis were investigated in the multivariate analysis. A multivariate logistic regression model was constructed with both backward and forward variable selection procedures based on the likelihood ratio test. Goodness of fit was assessed with the Hosmer-Lemeshow test. The p-values were two-sided. The type I error was set at 0.05.

Dealing with missing data was as follows: complete case analysis if less than 5%, multiple imputation by equation chained if between 5% and 30% and variable excluded if more than 30%. In case of multiple imputation by equation chained, data was imputed using an imputation model repeated 10 times. An analysis model was fit in each of the 50 imputed datasets separately and these 50 datasets were therefore pooled and gave overall sets of estimates and corresponding standard errors.

The model was internally validated using 200 bootstrap samples. Within each bootstrap sample, we refitted the model and compared the apparent performance in the bootstrap sample with the test performance (applying the refitted model to the original data). Optimism was calculated as the difference between the bootstrap performance and the test performance. We repeated 100 times the process, averaged the estimate of optimism and subtracted the value from the apparent performance to obtain an optimism-corrected estimate of performance. The test reflects therefore how robust the findings are when modest changes are made to the population; they are presented with an optimism corrected AUC.

To determine the prognosis score from the multivariate model, continuous data have been categorized and a threshold was determined based on the distribution of patients and a final scoring system was constructed based on the regression coefficient. Analyses were undertaken with R 3.1.1 statistical software using the packages stepwise, pROC, MKmisc, mice and rms (R foundation for Statistical Computing Vienna, Austria).

## Results

### Clinical characteristics of patients on initial presentation

156 adult consecutive patients had a diagnosis of aHUS and were treated with PE. About 50% had digestive signs, mainly abdominal pain (31%) and diarrhoea (29%). Half of patients exhibited neurologic impairment, ranging from headache (18%) to seizure (11%) and coma (9%). Mean arterial pressure (MAP) was high (107 [93–118] mmHg). At diagnosis, features of infection were frequent (48%) [15]. However, an infectious agent was identified in only 24% of cases. Median platelet count was 56 [30–103] X 10^3^/μL. Renal failure on admission was usually severe with a mean serum creatinine level at diagnosis of 4.1 [2.3–7] mg/dL. Dialysis was required in 54% of cases. Median ADAMTS13 activity was 44% [33–65]. A search for a complement abnormality was performed in 42 patients and one or more mutations were identified in 14 patients on CFH (5 patients), MCP (4 patients), CFI (3 patients), CFB and C3 genes (1 patient each). Detailed genetic abnormalities were reported previously [5]. Two additional patients had antibodies directed against CFH protein. In total, 38% of patients who were explored had complement abnormalities (Table 1).

**Table 1:** Characteristics of patients at baseline.

Variables		Cohort (n = 156)	CKD (n = 66)	No CKD (n = 44)	*P-value*
**Female gender**		107 (68.6%)	46 (69.7%)	29 (65.9%)	.68
**Age (years)**		46 [31–66]	45 [34–63]	46 [28–63]	.62
**Ethnicity**	Caucasian	144 (92.3%)	62 (93.9%)	43 (93.7%)	.75
	African	9 (5.8%)	2 (3%)	1 (2.3%)	
**Comorbidity**	Arterial hypertension	44 (28.2%)	23 (34.9%)	9 (20.4%)	.16
	Diabetes mellitus	10 (6.4%)	4 (6.1%)	4 (9.1%)	.83
	BMI	23 [21–26]	23 [21–25]	24 [21–25]	.64
	Tobacco addiction	40 (25.6%)	17 (25.8%)	9 (20.4%)	.48
	Cardiopathy *	25 (16%)	8 (12.1%)	7 (15.9%)	.75
	CTD**	18 (11.5%)	13 (19.7%)	1 (2.3%)	.007
	History of TMA	10 (6.4%)	6 (9.1%)	2 (4.6%)	.47
**Arterial pressure (mmHg)**	Systolic	150 [130–170]	160 [146–180]	140 [116–152]	< .001
	Diastolic	80 [70–94]	90 [80–100]	80 [70–90]	< .001
	Mean	107 [93–118]	113 [103–123]	97 [89–113]	< .001
**Digestive symptoms**	Overall	80 (51.3%)	36 (54.5%)	23 (53.5%)	> .9
	Diarrhoea	46 (29.5%)	21 (31.8%)	14 (32.6%)	> .9
	Abdominal pain	49 (31.4%)	24 (36.4%)	15 (34.9%)	> .9
**Neurologic symptoms**	Overall	76 (48.7%)	31 (47%)	18 (40.9%)	.56
	Headache	28 (17.9%)	13 (19.7%)	5 (11.4%)	.3
	Confusion	18 (11.5%)	13 (19.7%)	10 (22.7%)	.81
	Seizure	18 (11.5%)	9 (13.6%)	5 (11.4%)	.78
	Coma	14 (8.9%)	7 (10.6%)	3 (6.8%)	.74
**Blood cell count**	Haemoglobin (g/dL)	8.7 [7.2–10]	8.3 [6.9–9.7]	9.4 [8.3–10.5]	.006
	Platelets (10^3^/μL)	56 [30–103]	84 [49–121]	41 [25–59]	< .001
	WBC (10^3^/μL)	9.3 [7–13.2]	8.9 [7.1–12.7]	9.7 [7–12.9]	.4
**Haemolysis**	LDH (xN)	4.5 [2.5–8]	3.6 [2.4–5.9]	6.3 [3.3–8.6]	.019
**Renal impairment**	Serum creatinine (mg/dL)	4.1 [2.3–7]	6.3 [3.7–8.8]	2.8 [1.4–4.1]	< .001
	Renal replacement therapy	85 (54.5%)	54 (81.8%)	10 (22.7%)	< .001
**ADAMTS13 (%)**		44 [33–65]	41 [30–62]	51 [40–68]	.08
**Infection**	Fever	26 (16.7%)	10 (15.1%)	12 (29.2%)	.09
	Suspected	76 (48.7%)	23 (34.9%)	31 (70.4%)	< .001
	Documented	37 (23.7%)	9 (13.6%)	15 (34.1%)	.02
**Complement exploration**	CH50 (%)	93 [69–111]	97 [67–115]	84 [68–100]	.14
	C3 (mg/L)	898 [715–1140]	866 [669–1138]	875 [702–1012]	.87
	C4 (mg/L)	225 [128–300]	254 [200–326]	179 [100–280]	.01
	Factor B (μg/L)	138 [110–167]	139 [116–165]	139 [109–179]	.86
	Factor H (μg/L)	110 [91–127]	113 [86–127]	100 [89–128]	.79
	Factor I (μg/L)	116 [102–131]	119 [105–133]	104 [91–135]	.16
	CD46 (μg/L)	700 [553–800]	700 [546–800]	700 [536–763]	.66
Search for genetic abnormalities*** **/ anti-CFH Abs**		42 (26.9%)	24 (36.4%)	12 (27.2%)	.41

*Included ischemic cardiopathy and congestive heart failure.

**Included Crohn’s disease (n = 5), Sjögren’s syndrome (n = 2), Hashimoto’s thyroiditis (n = 2), rheumatoid arthritis (n = 5), ANCA (anti-neutrophil cytoplasm antibodies)-mediated vasculitis (n = 1), Mc Duffie vasculitis (n = 1), unclassified cutaneous vasculitis (n = 1) and autoimmune thrombocytopenia (n = 1).

***Included search for CFH, CFI, CFB, MCP and C3 component of complement pathway.

Values are expressed in percentage of subjects or in median numbers [interquartile range]. Statistical comparisons were made using the Wilcoxon two-sample test for continuous variables and the Chi-square test or the Fisher’s exact test was used to compare binary data. Abbreviations: CKD, chronic kidney disease; TMA, thrombotic microangiopathy; CTD, connective tissue disease; WBC, white blood cells; LDH, Lactate dehydrogenase; ADAMTS13, a disintegrin and metalloproteinase with thrombospondin type 1 repeats-13rd member.

### Clinical outcome

14 patients (9%) died during their hospital stay from aHUS or treatment complications. Renal prognosis was poor. After a 1-year follow-up, 66 patients (42%) developed mild to severe CKD (CKD group) (eGFR < 60mL/min/1.73m^2^ [stage 3a to 5]), whereas the remaining patients (no CKD group) recovered a normal (eGFR > 90 mL/min/1.73m^2^ [stage 1], N = 15 patients) or mildly altered (eGFR = 60 to 90 mL/min/1.73m^2^ [stage 2], N = 29 patients) renal function. The remaining patients died from another cause (5 cases) or were lost to follow-up (41 cases). At 5 years, 21 patients (13%) were undergoing haemodialysis and 13 (8%) were renal transplanted.

### Risk factors for chronic kidney disease

We compared patients who developed a CKD (eGFR < 60 ml/min/1.73m^2^; CKD group) to those who improved renal function (eGFR ≥ 60 ml/min/1.73m^2^; no CKD group). In univariate analysis, except with a history of connective tissue disease (*p* = .*007*), no comorbidity was associated with a poor renal outcome. A documented infection was more frequently observed in patients with no CKD (*p* = .*02*), but the nature of the micro-organisms was comparable between groups. Patients with CKD presented with a higher blood pressure on admission (*p <* .*001*). The severity of renal failure on diagnosis was highly predictive of CKD. Indeed, serum creatinine level on diagnosis was higher (*p <* .*001*) in patients with CKD, with more dialysis requirement (*p <* .*001*). Patient with no CKD presented a lower platelet count on diagnosis (*p <* .*001*) (Table 1).

Patients with CKD experienced more relapses (*p* = .*007*). Time to platelet count recovery was comparable between both groups but the length of hospitalization was longer in patients with CKD (*p* = .*002*) (Table 2). As expected, 5-year ESRD was more frequent in patients with CKD (*p <* .*001*), with more kidney transplantation at five years (*p <* .*001*).

**Table 2:** Treatment and outcome.

Variables		CKD (n = 66)	No CKD (n = 44)	*P-value*
**Treatment**	PE	53 (80.3%)	36 (81.8%)	.9
	Number of PE / patient	11 [5–20]	8 [3–14]	.32
	Steroids	39 (59.1%)	27 (62.8%)	.84
**Follow-up**	Death	4 (6.1%)	1 (2.3%)	.64
	Time to platelet count recovery (d)	11 [5–33]	6 [6–16]	.25
	Length of stay (d)	31 [21–53]	21 [15–27]	.01
	eGFR (mL/min/m^2^)	16 [5–42]	82 [73–95]	< .001
	Relapse	13 (19.7%)	1 (2.3%)	.007
	Kidney transplantation	13 (30.9%)	0 (0%)	< .001

Values are expressed as percentage of subjects or as median [interquartile range]. Statistical comparisons were made using the Wilcoxon two-sample test for continuous variables and the Chi-square test or the Fisher’s exact test was used to compare binary data. Abbreviations: CKD, chronic kidney disease; PE, plasma exchange. d, day; eGFR, estimated glomerular filtration rate.

In the multivariate analysis model, when considering data on admission, potential risk factors of CKD were a more severe renal dysfunction, a higher MAP and a higher platelet count. When we performed a sensitivity analysis with missing data imputed, the same risk factors were observed (not shown). An increase in serum creatinine by 0.55 mg/dL was associated with an increased risk of CKD (OR = 1.29 [1.14–1.50], *p <* .*001*). An increase in MAP by 10 mmHg was also associated with an increased risk of CKD (OR = 1.63 [1.22–2.35], *p* = .*003*). An increase in platelet count by 10 X 10^3^/μL was associated with a tenfold-increased risk of CKD (OR = 1.22 [1.08–1.43], *p* = .*008*). The Hosmer Lemeshow goodness of fit test was non-significant (*p* = .*58*), consistent with an accurately specified logistic regression model.

**Fig 1:**
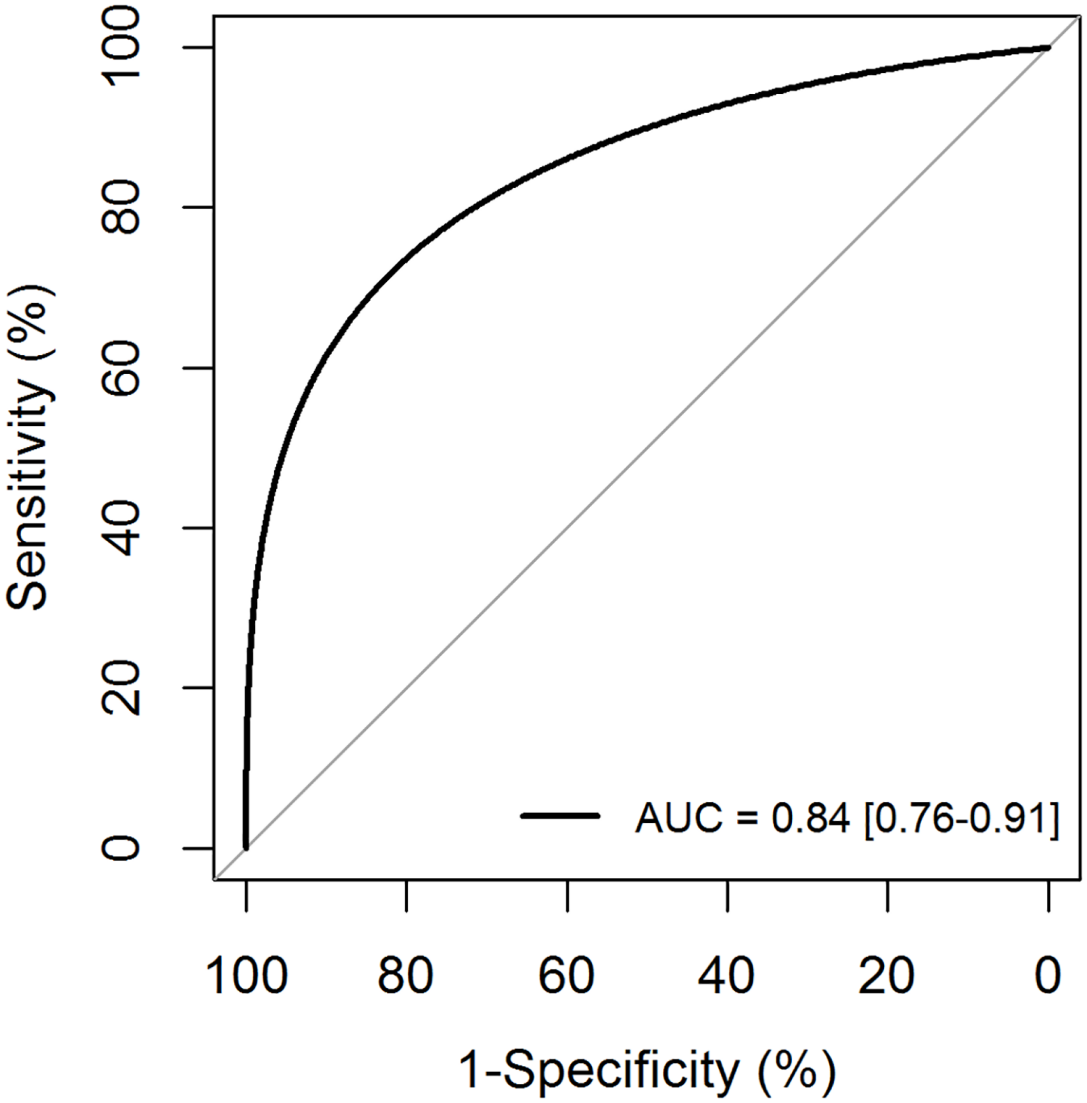
ROC curve.

**Fig 2:**
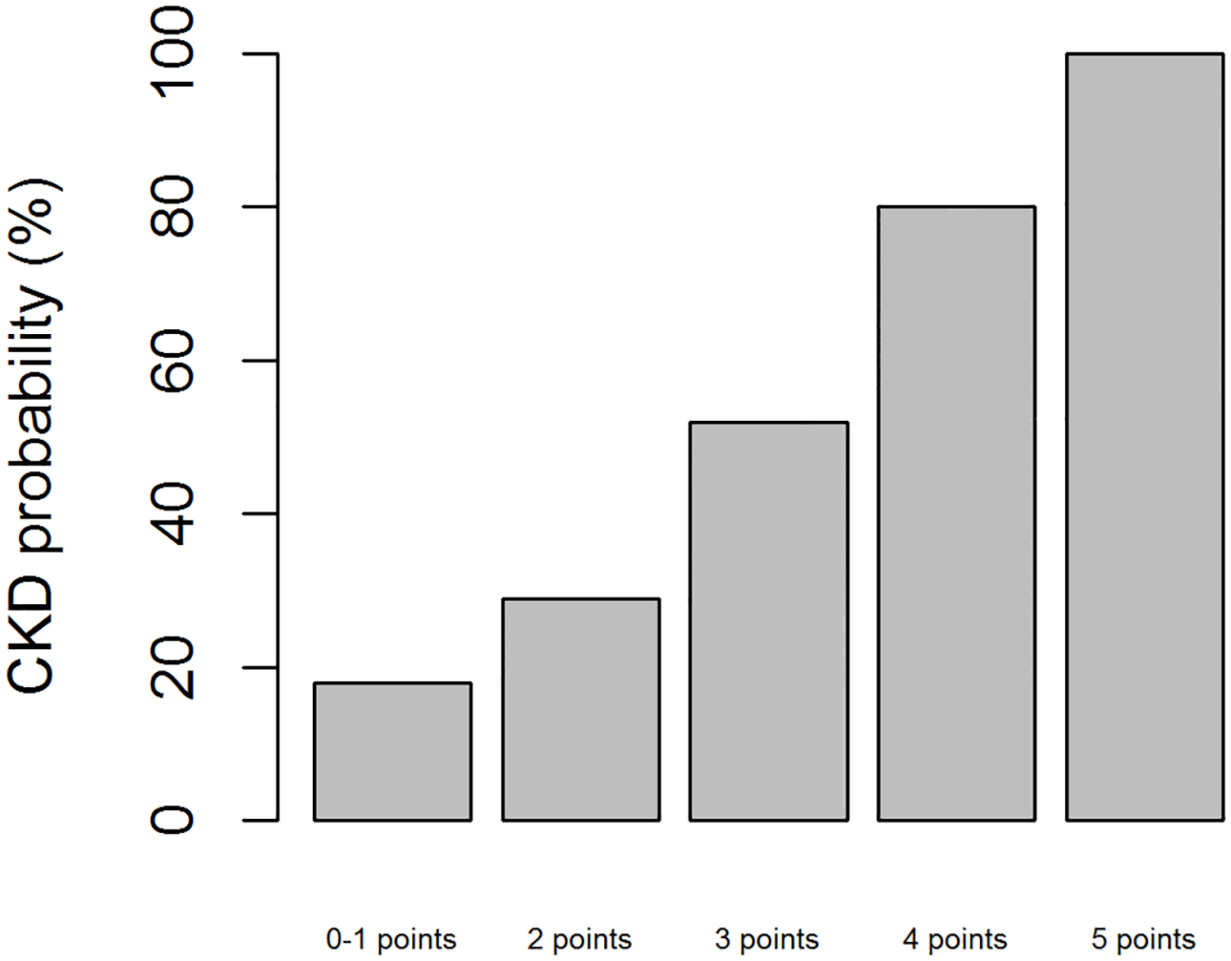
Probability of CKD according to the prognostic score.

A model to predict CKD was constructed using these 3 parameters and showed a good discriminative ability (AUC = .84) (Fig 1). The CKD score was based on the regression coefficient of the 3 predictors in the multivariate model. The score ranged from 0 to 5, with 4 categories for serum creatinine at admission, 2 categories for platelet count and 2 categories for MAP (Table 3). The corresponding risk for 1-year CKD ranges from 18 to 100% (Fig 2). The internal bootstrap validation indicates only modest over-fitting (optimism corrected AUC of .82), confirming the robustness of our results.

**Table 3:** CKD score.

**Serum creatinine (mg/dL)**	
0–1	0
1.1–3.39	1
3.4–5.64	2
> 5.65	3
**Platelets (10**^**3**^**/μL)**	
0–59	0
> 60	1
**Mean arterial pressure (mmHg)**	
0–105	0
> 106	1

### Characteristics of patients with a favourable CKD score (< 2)

Thirty-six patients (23%) had a CKD score < 2. This group of patients was characterized by a favourable renal outcome after PE, with only 3 (8.3%) cases of mild CKD (1-year eGFR 54, 55 and 59 mL/min/1.73m^2^), and 1 relapse at 1 year. As expected, renal involvement at presentation was mild (median serum creatinine level 1.1 mg/dL [range, 0.9–1.9]), and dialysis was required in only 4 cases (11%). MAP was 95 mmHg [range, 86–100], whereas thrombocytopenia was severe (27 X 10^3^/μL [range, 8–41]). Interestingly, among 10 patients investigated for complement genetic abnormalities, 2 displayed a mutation (CFH and MCP). On the opposite, a majority of non-survivors (74%) had a CKD score > 2.

## Discussion

Accurate diagnostic criteria of aHUS remain controversial. This limitation for an accurate management of aHUS patients arises whereas highly efficient targeted therapies are now available [8]. While TTP diagnosis was rendered easier by the identification of a severe ADAMTS13 deficiency [16], no reliable biological markers allow a rapid and accurate diagnosis of aHUS. A detectable ADAMTS13 activity is a prerequisite for this diagnosis [1,17]. However, within patients with features of aHUS (i.e., TMA patients with renal failure, a detectable ADAMTS13 activity and no associated STEC), distinct diseases with specific pathophysiological mechanisms, therapeutic modalities and prognosis may be further individualized. Here, our results provide evidence that according to clinical presentation, prognosis of renal function in patients with a diagnosis of aHUS and treated with PE before the era of the complement blocker eculizumab is variable, ranging from complete renal function recovery following TMA episode to ESRD. Therefore, ADAMTS13 activity alone (*i*.*e*., a detectable activity) is not sufficient to identify patients at risk of CKD who would benefit from complement blockers.

We report that severe renal impairment, high blood pressure and a mildly decreased platelet count in aHUS at diagnosis are associated with significant 1-year renal impairment. From these observations, we developed a reliable clinical score to predict patients at risk of CKD that should help identifying aHUS patients who might not benefit from PE, and in whom complement blockers frontline might be more efficient. Our score is able to predict a complete renal function recovery of aHUS treated with PE in patients with a score of 0 (*i*.*e*., with a serum creatinine level < 1.1 mg/dL, a platelet count < 60 X 10^3^/μL and a MAP < 106 mmHg), whereas patients with a score of 5 (*i*.*e*., with a serum creatinine level > 5.65 mg/dL, a platelet count > 60 X 10^3^/μL and a MAP ≥ 106 mmHg) experience CKD in 100% of cases. Therefore, one could suggest that in patients with a score > 2, PE poorly prevents CKD with a disadvantageous risk/benefit balance [18]). In line with these statements, we found that non-survivors mostly had a high CKD score (> 2) consistent with a more severe disease on diagnosis, which suggests that our score could also reliably identify patients at higher risk of death. A strength of our score is that it is based on clinical features easily assessable on diagnosis, allowing to evaluate renal prognosis in real time, which may assist in clinical decision making to provide the more adapted therapy (*i*.*e*., PE versus complement blockers) [8,11].

In line with our results, previous studies defined prognostic factors of CKD in TMA [19–21]): no PE procedures [21], a pre-existing nephropathy, a high MAP, and severe renal involvement on diagnosis [22,23]. However, the main limitation of these studies was their retrospective design. Moreover, they were conducted before the era of ADAMTS13 assessment, so that patient subgroups were heterogeneous. Here, although the retrospective design of our study, our patients had a similar evaluation, they were all selected on the basis of ADAMTS13 activity assessed in a reference laboratory, and cases associated with confounding factors were excluded, providing accuracy to our results.

An interesting finding of our work is that patients with a CKD score < 2 display features consistent with TTP diagnosis: no or mild renal involvement, a more pronounced thrombocytopenia, a normal or mildly increased MAP, and a favourable prognosis with daily PE despite a detectable or normal ADAMTS13 activity with standard assays. Whether subtle alterations in ADAMTS13 function, that may not be assessable by current assays, are involved in the pathophysiology of this subset of TMA remains to be stated, and further works are now needed to identify the specific pathophysiological mechanisms in this context. On the other hand, 20% of these patients who were explored for genetic abnormalities of the complement system were found to have a mutation, in accordance with a previous report [24]. Moreover an infectious process, which could have triggered the disease, preceded aHUS in half of cases [4]. An interesting hypothesis from our results is that renal failure in patients with a suspected or documented infection may result more directly from a strong, infection-mediated, endothelial injury. On the opposite, in patients with no infectious triggering factor, it is likely that complement has a more prominent role, accounting for a poorer renal prognosis with only PE. In line with this hypothesis, the high prevalence of infectious events in our series of patients, also reported by others [15], may account for the lower prevalence of complement abnormalities as compared to this reported from patients with no infection [5].

Our study has limitations. The retrospective design accounts for missing data during follow-up, particularly proteinuria, which is an important and known marker for CKD. Moreover, we were not able to externally validate the CKD score, although a bootstrap validation was successful [25]. Consequently, our score now requires prospective validation before it can assist in clinical decision making in daily practice.

In conclusion, we identified the magnitude of renal dysfunction, high blood pressure and a higher platelet count on diagnosis as predictors of unfavourable renal outcome in aHUS. We also established a clinical score able to predict persistent renal failure that should help identifying patients suitable for a rapid treatment with complement blockers.

## Supporting information

S1 Table KDIGO classification of chronic kidney disease. (DOCX)
