# Supplementary material for: Predictive features of chronic kidney disease in atypical haemolytic uremic syndrome
Source: PLoS One. 2017 May 18;12(5):e0177894. doi: 10.1371/journal.pone.0177894 (PMC5436831; doi:10.1371/journal.pone.0177894)
Supplement: S1 Table — (DOCX) [file pone.0177894.s001.docx]

Supplement Table 1. KDIGO classification of Chronic Kidney Disease

| **GFR Categories** | | **eGFR (mL/min/1.73m^2^)** |
| --- | --- | --- |
| G1 | Normal or High | ≥ 90 |
| G2 | Mildly decreased | 60 - 90 |
| G3a | Mildly to moderately decreased | 45-59 |
| G3b | Moderately to severely decreased | 30-44 |
| G4 | Severely decreased | 15-29 |
| G5 | Kidney failure | < 15 |
